# Share of Adult Suicides After Recent Jail Release

**DOI:** 10.1001/jamanetworkopen.2024.9965

**Published:** 2024-05-10

**Authors:** Ted R. Miller, Lauren M. Weinstock, Brian K. Ahmedani, Nancy N. Carlson, Kimberly Sperber, Benjamin Lê Cook, Faye S. Taxman, Sarah A. Arias, Sheryl Kubiak, James W. Dearing, Geetha M. Waehrer, James G. Barrett, Jessica Hulsey, Jennifer E. Johnson

**Affiliations:** 1 National Capital Region Center, Pacific Institute for Research and Evaluation (PIRE), Beltsville, Maryland; 2 Curtin University School of Public Health, Beltsville, Maryland; 3 Department of Psychiatry and Human Behavior, Alpert Medical School of Brown University, Providence, Rhode Island; 4 Center for Health Policy and Health Services Research, Henry Ford Health System, Detroit, Michigan; 5 School of Counseling, Walden University, Silver Spring, Maryland; 6 Complex Health Solutions, Behavioral Health and Wellness, CareSource, Dayton, Ohio; 7 Harvard Medical School, Cambridge, Massachusetts; 8 Cambridge Health Alliance, Cambridge, Massachusetts; 9 Schar School of Policy and Government, Center for Advancing Correctional Excellence, George Mason University, Fairfax, Virginia; 10 Butler Hospital, Providence, Rhode Island; 11 Center for Behavioral Health and Justice, Wayne State University School of Social Work, Detroit, Michigan; 12 Department of Communication, Michigan State University, East Lansing; 13 Cambridge Police Department, Cambridge, Massachusetts; 14 Addiction Policy Forum, Bethesda, Maryland; 15 Charles Stewart Mott Department of Public Health, Michigan State University, East Lansing

## Abstract

**Importance:**

Although people released from jail have an elevated suicide risk, the potentially large proportion of this population in all adult suicides is unknown.

**Objective:**

To estimate what percentage of adults who died by suicide within 1 year or 2 years after jail release could be reached if the jail release triggered community suicide risk screening and prevention efforts.

**Design, Setting, and Participants:**

This cohort modeling study used estimates from meta-analyses and jail census counts instead of unit record data. The cohort included all adults who were released from US jails in 2019. Data analysis and calculations were performed between June 2021 and February 2024.

**Main Outcomes and Measures:**

The outcomes were percentage of total adult suicides within years 1 and 2 after jail release and associated crude mortality rates (CMRs), standardized mortality ratios (SMRs), and relative risks (RRs) of suicide in incarcerated vs not recently incarcerated adults. Taylor expansion formulas were used to calculate the variances of CMRs, SMRs, and other ratios. Random-effects restricted maximum likelihood meta-analyses were used to estimate suicide SMRs in postrelease years 1 and 2 from 10 jurisdictions. Alternate estimate was computed using the ratio of suicides after release to suicides while incarcerated.

**Results:**

Included in the analysis were 2019 estimates for 7 091 897 adults (2.8% of US adult population; 76.7% males and 23.3% females) who were released from incarceration at least once, typically after brief pretrial stays. The RR of suicide was 8.95 (95% CI, 7.21-10.69) within 1 year after jail release and 6.98 (95% CI, 4.21-9.76) across 2 years after release. A total of 27.2% (95% CI, 18.0%-41.7%) of all adult suicide deaths occurred in formerly incarcerated individuals within 2 years of jail release, and 19.9% (95% CI, 16.2%-24.1%) of all adult suicides occurred within 1 year of release (males: 23.3% [95% CI, 20.8%-25.6%]; females: 24.0% [95% CI, 19.7%-36.8%]). The alternate method yielded slightly larger estimates. Another 0.8% of adult suicide deaths occurred during jail stays.

**Conclusions and Relevance:**

This cohort modeling study found that adults who were released from incarceration at least once make up a large, concentrated population at greatly elevated risk for death by suicide; therefore, suicide prevention efforts focused on return to the community after jail release could reach many adults within 1 to 2 years of jail release, when suicide is likely to occur. Health systems could develop infrastructure to identify these high-risk adults and provide community-based suicide screening and prevention.

## Key Points

**Question:**

What proportion of US adults who died by suicide spent at least 1 night in jail shortly before their death?

**Findings:**

In this cohort modeling study involving nearly 7.1 million US adults released from incarceration in 2019, nearly 20% of suicides occurred among those who were released from jail in the past year and 7% were by those in their second year of jail release.

**Meaning:**

Findings of this study suggest that focused suicide prevention efforts could reach a substantial number of adults who were formerly incarcerated within 2 years, when death by suicide is likely to occur.

## Teaser

This cohort study estimates the risk of suicide among US adults returning to the community after release from incarceration.

## Introduction

Unlike most high-income countries, the US has a 2-tiered incarceration system. All people in state and federal prisons already received a trial and conviction, primarily of felonies, and are serving their sentence. In contrast, jails, which are operated by local governments, primarily detain unconvicted people awaiting arraignment or held over for trial. Just 35% of people released from jail serve sentenced time.^1^ Those sentenced to jail receive short sentences (<1 year), primarily for low-level felonies or violating terms of sentenced supervision in the community. Individuals who are unsentenced often spend only a few days in jail. In 2019, US jails had 10 322 570 admissions, with many people admitted multiple times, and a mean daily census of 734 470.^2^ The weekly turnover rate was 53%.^1^ Rapid return from jail to the community is the norm.

People in jail have a high annualized suicide rate (48 per 100 000 people) and a standardized mortality ratio (SMR) of 2.2 compared with demographically similar US adults.^3^ Growing global literature suggests an even higher suicide rate in the year after release from incarceration; meaning that more suicides per day occur after release than during jail time.^4,5^ Only 1 study with high-quality death registration, however, is specific to US jails: among Philadelphia Medicaid beneficiaries who died by suicide between 2003 and 2018, 25% had a history of Philadelphia county jail incarceration.^6^

In the US, no data exist on suicide risk during an actionable period of 1 to 2 years after jail release. This cohort modeling study aimed to estimate the percentage of adults who died by suicide within the year or 2 years after jail release who could have been reached if the jail release triggered community suicide risk screening and prevention efforts. It applied meta-analytic and epidemiologic modeling to published cohort studies of suicide after release from prison or incarceration in the US or abroad. It also examined the size of the adult jail population and modeled the risk of suicide within 1 to 2 years after a jail stay in this population compared with adults with no recent incarceration and the portion of adult suicides during that vulnerable period. This study built models from publicly available aggregate counts; it used no unit record data.

## Methods

In accordance with the Common Rule, this cohort study was exempt from ethics review and informed consent requirement because it was not considered human participant research. We followed the Strengthening the Reporting of Observational Studies in Epidemiology (STROBE) reporting guideline.

### Data Sources and Calculations 

#### Jail Counts

Every year, federal reports provide the mean daily population in US jails and prisons^2^ and annual counts of admissions.^1^ We computed mean length of jail stay by dividing annual jail admissions^1^ by 365 times the mean daily jail census.^2^

Many people discharged from jail are readmitted in the same year. Given that arrest counts have been virtually identical to jail admission counts,^7^ dividing annual admissions by annual arrests per person arrested approximates the number of people jailed annually. The National Survey on Drug Use and Health variable NoBookYr2 reported annual arrests per person arrested and booked during the past 12 months from 2015 to 2018.^8^

#### Suicide Risk and Counts 

Federal reports count suicide deaths that occur, separately, in jail and in prison.^3^ Multiplying the death counts by 100 000 divided by the mean daily population yields a crude mortality rate (CMR) per 100 000 person-years by setting.

Federal reports calculate the suicide CMR for cohorts of US adults matched to the jail and prison populations by age, sex, and race and ethnicity. Dividing the suicide CMR for those in jails or prisons by the demographically matched suicide CMR for US adults with no recent incarceration yields the federally reported SMRs by setting. Meanwhile, dividing the suicide CMR for those in jails or prisons by the unadjusted national rate for adults not incarcerated in the past year yields a relative risk (RR) of suicide death.

As a proxy for suicide RRs after jail release, we used RRs after prison release, with emphasis on studies from countries that house unconvicted people in prison. To identify linked data on deaths of people discharged from incarceration, we started with 5 systematic reviews identifying studies published through October 2021.^4,5,9,10,11^ We updated from there by scanning all studies that cited the systematic reviews from 2021 onward; the 191 summaries from a Google Scholar search from 2021 through January 2024 using the terms suicide, linkage, (incarcerat* or jail or prison), post-release, and *(death or mortality)*; and first-screen hits when we used Google Scholar or PubMed to retrieve each relevant study.

The systematic reviews focused on lifetime rather than acute suicide risk. Seeking acute risk data and recent studies, 1 of us (T.R.M.) read all studies in the systematic reviews and the 23 potentially relevant studies that we identified. One of us (T.R.M.) also verified the systematic review coding and the quality of study CMR or SMR computations.

Included studies had to provide a demographically matched SMR or the data to compute an SMR using the Centers for Disease Control and Prevention’s Web-Based Injury Statistics Query and Reporting System (WISQARS) and at least 2 of the following: a CMR, a suicide count, and person-years of exposure. We included only 1 study per jurisdiction, giving preference to studies that provided CMRs at year 1 or year 2 after release, with next priority going to studies that covered the shortest duration beyond 2 years. We excluded studies involving juveniles, subpopulations, or fewer than 40 suicide deaths over 2 years after release. For supplemental analyses, we also sought CMRs per person-year during incarceration.

### Statistical Analysis

Using Excel version 2401 (Microsoft Corp), we applied published formulas for the variance of CMRs and SMRs and Taylor expansion formulas for the variance of other ratios,^12,13^ and then we ran random-effects restricted maximum likelihood (RE-ML) meta-analyses using RStudio 4.3.2 (RStudio Team). We built 10 jurisdictional estimates from 8 adult incarceration studies^14,15,16,17,18,19,20,21^ identified in the 2023 meta-analysis by Janca et al^5^ plus 3 studies we identified.^22,23,24^ All studies were dominated by decades-old data. All except 3 studies^14,20,24^ gave partial information on suicide timing in the 2 years after release. We used ratios of suicide CMRs in postrelease year 1 and year 2 and narrow assumptions or calculations (eTable 1 in Supplement 1) to adjust study data from diverse time periods to estimates in postrelease year 1 and year 2. We calculated the CMR of demographically similar people without recent incarceration for 1 study^24^ (eTable 2 in Supplement 1).

We developed estimated counts of suicide deaths after incarceration from the meta-analysis and 4 sensitivity analysis sets of studies with different strengths and limitations. For each set, we multiplied the postrelease SMR by the 2019 US CMR for nonincarcerated adults to estimate a postrelease CMR for the US. The number of postrelease suicides equaled the released person count times the CMR. The study sets comprised (1) our primary estimate; (2) a study from Ontario, Canada^17^; (3) the SMR for releases in England and Wales^23^; (4) a consolidated estimate from separate computations by sex; and (5) the meta-analysis by Janca et al.^5^

First, we estimated suicide SMRs in postrelease years 1 and 2 using meta-analytic RE-ML pooling of SMRs from 10 jurisdictions. Variance for CMRs and SMRs were computed using standard formulas.^12^ Six of these high-quality studies^16,17,18,19,20,21,22^ included unconvicted individuals, 4 were from North America,^14,17,23,24^ and all were adjusted to cover equal periods.

Second, a study by Kouyoumdjian et al^17^ linked deaths to incarceration releases in 2000 throughout the Canadian province of Ontario and provided a detailed time track for suicide deaths in the year after release and a mean rate for the following 2 years. Its North American data matched US jail data on the percentage of females and on 63% vs 65% of nondeported individuals who were released unconvicted.

Third, the SMR from 1 year of tracking data for all people who were released from incarceration in England and Wales during 2000 to 2002, of which 35% were individuals who were unconvicted or released to the community on sentencing.^18^ We corrected the published SMR for both sexes combined, which erroneously had been computed by averaging community CMRs for nonincarcerated persons by sex rather than by weighting them to match the sex distribution of the incarcerated population. The unconvicted persons were reasonably well represented, and tracking was for exactly 1 year.

Fourth, we developed a consolidated estimate from separate computations for males and females using SMR estimates for postrelease year 1 using RE-ML meta-analytic pooling of data from 6 adult incarceration studies. We matched this estimate to the US release distribution by sex. Five studies^16,18,20,21,22^ included people who were released unconvicted, all studies were adjusted to cover 1 year, but only 1 study^23^ was from North America.

Fifth, the 2023 meta-analysis of 11 studies by Janca et al^5^ provided an independently developed, peer-reviewed SMR estimate. The meta-analysis gave preference to SMRs over more years rather than 1 to 2 years, diluting the higher risk soon after release; thus we considered its estimates weak. The between-study heterogeneity was high for duration and population, with juvenile detention and studies of males alone included. Two studies had SMR calculation errors.^18,25^

To calculate the suicide CMR among adults who were not incarcerated, we subtracted the number of suicide deaths during and after a jail or prison stay from the WISQARS adult suicide count and the number of adults incarcerated from its population count.^26^ The CMR equaled 100 000 times suicide count divided by population count.

We developed another estimate using a distinctly different ratio method (hereafter referred to as ratio method) and meta-analysis of the RR of suicide death in the year after release vs while incarcerated. For example, in a UK study by Phillips and Roberts,^27^ the RR was 2.55 (CMR of 212 in the year after release divided by CMR of 83 per year incarcerated). Except with the Phillips and Roberts^27^ study, we used online counts of inmates and suicide deaths during incarceration^3^ to compute the ratios. We generated 8-study RE-ML meta-analytic RR estimates using this approach for postrelease year 1 and year 2. We multiplied the mean RRs by the 2019 US CMRs while in jail and in prison^3^ to estimate the postrelease CMRs. The final estimates were independent of the suicide rate in the general population. Half of the estimates were from North America. The CMR estimates while incarcerated, however, were not always definitive or perfectly matched. Data analysis and calculations were performed between June 2021 and February 2024.

## Results

This cohort modeling study included 2019 estimates for 7 091 897 adults (2.8% of US adult population; 76.7% males and 23.3% females) who were released from incarceration at least once, typically after brief pretrial stays. Table 1 and Table 2 describe the 10 studies that we analyzed and the meta-analytic consolidation of their data, including the CMRs and SMRs in year 1 (Table 1) and in the first 2 years combined (Table 2) after release from incarceration as well as the risk of suicide after release vs during incarceration (eTables 3 and 4 in Supplement 1 provide corresponding SEs and 95% CIs). For example, the 10 studies linked mortality data from more than 1 million people for 1 year after incarceration, finding 1409 suicide deaths. Among the 10 studies, the CMR range in the year after incarceration was 42.51 to 361.37 per 100 000 person-years (Table 1); across the 2 years after release, the CMR range was narrower: 42.75 to 242.93 per 100 000 person-years (Table 2).

**Table 1.** Meta-Analytic Data on Suicide in Year 1 After Incarceration Release

Source	Place of incarceration	No. of people released	Release dates	Periods reported	CMR per 100 000 person-years, postrelease year 1	SMR, postrelease year 1	No. of suicides, postrelease year 1	Person-years at risk, postrelease year 1
Binswanger et al,^14^ 2007	Washington, US	30 327	July 1999-December 2003	14 d, 2 y	94.05	4.57	27	28 525
Cunningham et al,^22^ 2022^a^	New Zealand	87 894	1998-2016	28 d, 1 y, 2 y, 9.4 y	361.37	16.04	315	87 169
Fitch et al,^23^ 2024^b^	North Carolina, US	474 892	2000-February 2020	28 d, 1 y, 2 y, 5.7 y	42.51	1.94	181	425 818
Haglund et al,^15^ 2014	Sweden	26 985	2005-2009	28 d, 2 y	235.65	21.02	78	33 100
Kariminia et al,^16^ 2007	New South Wales, Australia	85 196	1988-2002	28 d, 6 m, 7.7 y	203.63	6.48	229	112 335
Kouyoumdjian et al,^17^ 2016	Ontario, Canada	46 442	2000	28 d, 1 y, 2 y	99.40	8.43	46	46 277
McNeeley et al,^24^ 2023	Minnesota, US	36 716	2010-2019	4.9 y	112.26	4.88	41	36 466
Pratt et al,^18^ 2006; corrected	England and Wales, UK	245 029	2000-2002	1 y	155.93	8.92	382	245 029
Stewart et al,^20^ 2004	Western Australia, Australia	9381	1994-1999	3.4 y	291.78	7.17	27	9245
Van Dooren et al,^21^ 2013	Queensland, Australia	42 015	1994-2007	1 y, 7.5 y	216.67	12.00	84	38 769
10 Studies combined^c^	NA	1 054 550	NA	NA	177.64	8.95	1409	1 062 733

Abbreviations: CMR, crude mortality rate; NA, not applicable; SMR, standardized mortality ratio.

^a^ Randomly rounded counts for years 1 and 2 were provided by Ruth Cunningham (email communication to Dr Miller, January 25, 2024).

^b^ Counts for years 1 and 2 were provided by Kate Vinita Fitch (email communication to Dr Miller, February 8, 2024).

^c^ Studies were combined using restricted maximum likelihood random-effects meta-analysis. The Figure and eTable 3 in Supplement 1 show SEs and 95% CIs for CMRs, SMRs, and RRs.

**Table 2.** Meta-Analytic Data on Total Suicides in Years 1 and 2 After Incarceration Release

Source	Place of incarceration	% Of inmates unconvicted	CMR per 100 000 person-years, during incarceration	Release dates	RR (CMR after release divided by CMR in custody) (95% CI)		CMR per 100 000 person-years, combined years 1 and 2	SMR, combined years 1 and 2	No. of suicides, combined years 1 and 2	Person-years at risk, combined years 1 and 2
					Year 1	Year 2				
Phillips and Roberts,^27^ 2019	UK	NA	83.00	Fiscal year 2018-2019	2.55 (2.51 to 2.60)	1.98 (1.94 to 2.02)	164.42^a^	NA	237	144 340
Binswanger et al,^14^ 2007	Washington, US	0	12.81	July 1999-December 2003	7.34 (–23.00 to 37.68)	5.46 (–10.63 to 21.55)	70.00	3.40	40	57 049
Cunningham et al,^22^ 2022^b^	New Zealand	47.2	83.73	1998-2016	4.32 (3.70 to 4.93)	2.90 (2.64 to 3.17)	242.93	10.42	423	174 124
Fitch et al,^23^ 2024^c^	North Carolina, US	0	9.80	2000-February 2020	4.34 (2.69 to 5.99)	4.36 (2.79 to 5.94)	42.75	1.93	327	764 900
Haglund et al,^15^ 2014	Sweden	0	NA	2005-2009	NA	NA	204.00	18.20	127	48 157
Kariminia et al,^16^ 2007	New South Wales, Australia	19.9	125.22	1988-2002	1.63 (1.57 to 1.69)	1.32 (1.28 to 1.35)	164.88	5.25	324	196 651
Kouyoumdjian et al,^17^ 2016	Ontario, Canada	62.7	48.65	2000	2.04 (1.50 to 2.58)	1.38 (1.16 to 1.61)	67.23	5.70	62	92 223
McNeeley et al,^24^ 2023	Minnesota, US	NA	12.76	2010-2019	8.80 (–7.54 to 25.14)	6.59 (–0.67 to 13.85)	84.13	3.66	61	72 558
Pratt et al,^18^ 2006; corrected	England and Wales, UK	35.0	82.78	2000-2002	1.88 (1.83 to 1.94)	1.51 (1.48 to 1.53)	124.66	7.13	611	489 976
Stewart et al,^20^ 2004	Western Australia, Australia	15.8	NA	1994-1999	NA	NA	236.11	5.80	44	18 762
Spittal et al,^19^ 2014 and Van Dooren et al,^21^ 2013	Queensland, Australia	19.5	NA	1994-2007	NA	NA	173.01	9.60	128	73 985
10 Studies combined^d^	NA	NA	NA	NA	NA	NA	138.52	6.85	2147	1 994 641
8 Studies combined^d^	NA	NA	NA	NA	2.70 (1.78 to 3.31)	2.12 (1.38 to 2.87)	NA	NA	NA	NA

Abbreviations: CMR, crude mortality rate; NA, not applicable; RR, relative risk; SMR, standardized mortality ratio.

^a^ The CMR in year 1 was 212, based on 153 suicides and 72 170 person-years at risk.

^b^ Randomly rounded counts for years 1 and 2 were provided by Ruth Cunningham (email communication to Dr Miller, January 25, 2024).

^c^ Counts for years 1 and 2 were provided by Kate Vinita Fitch (email communication to Dr Miller, February 8, 2024).

^d^ Studies were combined using restricted maximum likelihood random-effects meta-analysis. The Figure and eTables 3 and 4 in Supplement 1 show SEs and 95% CIs for CMRs, SMRs, and RRs.

One study^27^ was included only for the RR analysis. The Figure shows the associated meta-analytic forest plots. For example, the SMR during the first year after release was 8.95 (95% CI, 5.54-12.36), with the study with the lowest SMR^23^ receiving the largest weight (10.43%) and the study with the highest SMR^15^ receiving the lowest weight (8.73%).

**Figure.**
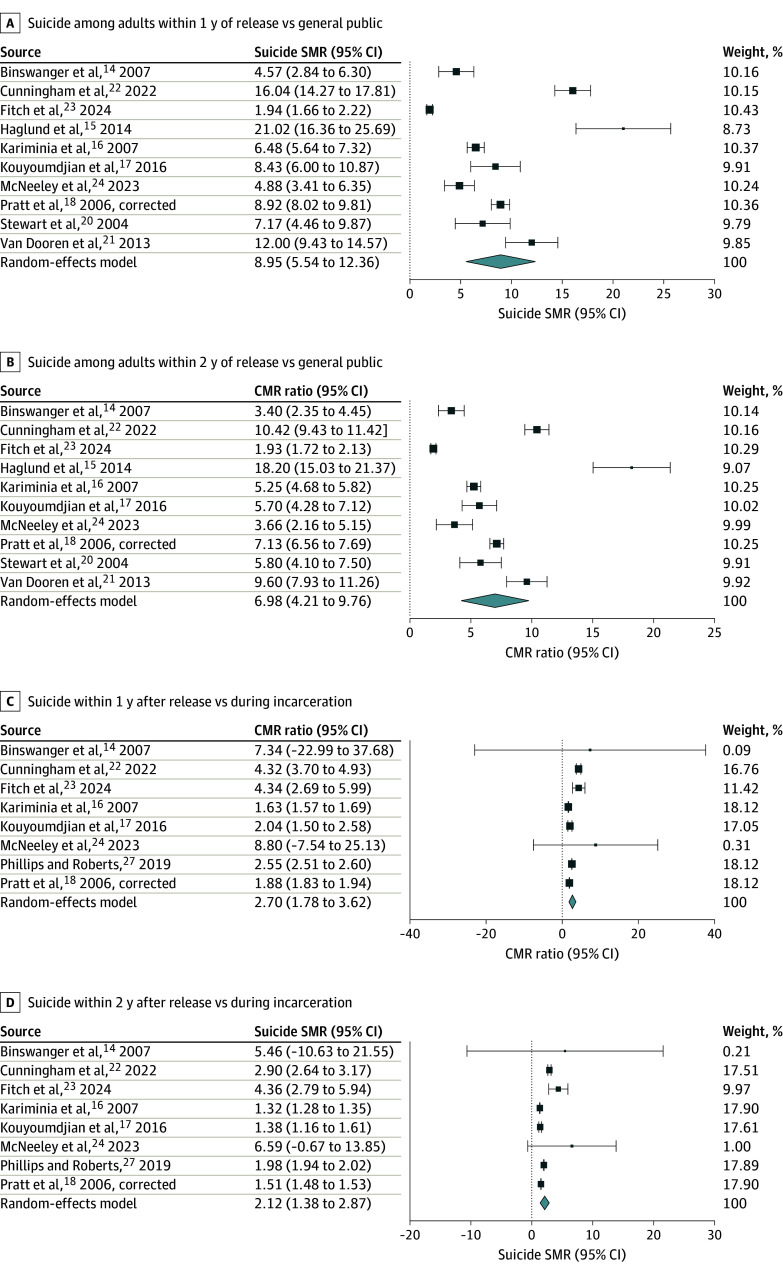
Meta-Analytic Estimates of Standardized Mortality Ratios (SMRs) for Suicide After Incarceration Release and Crude Mortality Rate (CMR) Ratios After vs During Incarceration Error bars represent 95% CIs; vertical dashed line at 0 represents the line of no effect; square size represents the study weight; and diamond represents the overall estimate from the meta-analysis, with the width representing the 95% CI of the point estimate.

### Jail Stay and Release

From 2015 to 2018, data from the National Survey on Drug Use and Health indicated that people who were arrested had a mean number of 1.456 arrests annually, with 73.9% arrested only once; 10% of these people were rearrested the next year.^23^ In 2019, 7 091 897 US adults (2.8% of adult population) were released from jail, with another 734 470 in jail on an average day.^2^ The mean jail stay (including both convicted and unconvicted individuals) was 24.7 days.

The best meta-analytic estimate (Table 3) showed that 9121 (95% CI, 7440-11 034) people died by suicide during the year after jail release (sensitivity analysis range, 8706-10 470 people; ratio method estimate, 9255 [95% CI, 6114-12 396] people). In 2019, 355 people died by suicide in US jails (Table 4).^2^

**Table 3.** Rate, Relative Risk (RR), and Percentage of Suicides in Adults Released from Incarceration or Not Recently Incarcerated, 2019

Data Source and calculation method	No. of adult suicides	Suicide rate per 100 000 adults	RR of adult suicides^a^	% Of adult suicides (95% CI)^b^	% Of adult population
**Postrelease year 1, estimate from 10-study meta-analysis; best estimate**					
Year 1 after jail release	9121	128.61	8.95	19.9 (16.2-24.1)	2.8
Year 1 after prison release	777	128.61	8.95	1.7 (1.4-2.0)	0.2
Age ≥18 y, no recent jail or prison	35 273	14.38	1.00	76.9 (73.0-80.9)	96.1
**Kouyoumdjian et al,**^17^ **2016 (Ontario, Canada)**					
Year 1 after jail release	8706	122.76	8.43	19.0 (13.5-24.5)	2.8
Year 1 after prison release	741	122.76	8.43	1.6 (1.1-2.1)	0.2
Age ≥18 y, no recent jail or prison	35 723	14.56	1.00	77.9 (71.9-83.9)	96.1
**Pratt et al,**^18^ **2006, corrected (England and Wales, UK)**					
Year 1 after jail release^c^	9098	128.29	8.92	19.8 (18.1-22.1)	2.8
Year 1 after prison release^c^	775	128.29	8.92	1.7 (1.5-1.9)	0.2
Age ≥18 y, no recent jail or prison	35 297	14.39	1.00	77.0 (75.2-78.9)	96.1
**6-study meta-analysis, calculated by sex**					
Year 1 after jail release	10 740	151.44	11.09	23.4 (15.1-31.7)	2.8
Year 1 after prison release	925	153.21	11.22	2.0 (1.3-2.7)	0.2
Age ≥18 y, no recent jail or prison	33 505	13.66	1.00	73.1 (64.7-82.0)	96.1
**11-study meta-analysis by Janca et al,**^5^ **2023; weak estimate not based on risk at year 1**					
Year 1 after jail release	7842	110.57	7.40	17.1 (12.2-21.2)	2.8
Year 1 after prison release	668	110.57	7.40	1.5 (1.0-1.8)	0.2
Age ≥18 y, no recent jail or prison	36 661	14.94	1.00	79.9 (76.2-85.3)	96.1
**Ratio method, year 1 estimate from 8-study meta-analysis**					
Year 1 after jail release	9255	130.50	9.09	20.2 (13.3-27.0)	2.8
Year 1 after prison release	708	117.31	8.18	1.5 (1.0-2.1)	0.2
Age ≥18 y, no recent jail or prison	35 207	14.35	1.00	76.8 (70.0-84.1)	96.1
**Postrelease year 2, estimate from 10-study meta-analysis; best estimate**					
Year 2 after jail release	12 483	92.64	6.98	27.2 (18.0-41.7)	5.3
Year 2 after prison release	1063	92.64	6.98	2.3 (1.5-3.6)	0.4
Age ≥18 y, no recent jail or prison	31 624	13.26	1.00	69.0 (53.8-78.9)	93.4
**Ratio method, year 2 estimate from 8-study meta-analysis**					
Year 2 after jail release	13 821	102.57	8.07	30.1 (19.6-40.7)	5.3
Year 2 after prison release	1058	92.20	7.26	2.3 (1.5-0.0)	0.4
Age ≥18 y, no recent jail or prison	30 292	12.70	1.00	66.0 (58.5-77.4)	93.4

^a^ The RR was computed using the variable age 18 years or older, no recent jail or prison as the denominator.

^b^ Percentage of adult suicides was computed from No. of adult suicides.

^c^ Included any readmission stays.

**Table 4.** Population, Suicides, Crude Mortality Rate (CMR), and Relative Risk (RR) of Suicide in US Adults by Sex, 2019^a^

Group	No. of adults	No. of adult suicides	CMR, %	RR of adult suicides^b^	% Of adult suicides (95% CI)^c^	% Of adult population^c^
**Total population**						
Year in jail, based on daily census	734 470	355	48.33	3.32	0.8 (0.8-0.8)	0.3
Year after jail release	7 091 897	10 740	151.44	11.09	23.4 (15.1-31.7)	2.8
Year in state prison custody	1 311 100	311	23.72	1.63	0.7 (0.7-0.7)	0.5
Year in federal prison custody	147 000	29	19.73	1.35	0.1 (0.1-0.1)	0.1
Year after prison release	603 844	925	153.21	11.22	2.0 (1.3-2.7)	0.2
Age ≥18 y, no recent jail or prison	245 352 967	33 168	13.52	1.00	72.3 (70.5-73.9)	96.1
Age ≥18 y	255 241 278	45 865	17.97	1.23	100 (100-100)	100
**Females only**						
Year in jail, based on daily census	110 735	42	38.11	6.91	0.4 (0.4-0.4)	0.1
Year after jail release	1 652 253	2342	141.73	25.13	24.0 (19.7-36.8)	1.3
Year in prison custody	135 735	17	55.00	9.78	0.2 (0.2-0.2)	0.1
Year after prison release	56 213	80	141.73	25.13	0.8 (0.7-1.3)	0.04
Age ≥18 y, no recent jail or prison	128 922 632	7251	5.62	1.00	74.4 (61.3-79.0)	98.5
Age ≥18 y	130 877 568	9752	7.45	1.35	100 (100-100)	100
**Males only**						
Year in jail, based on daily census	623 735	313	50.15	2.23	0.9 (0.9-0.9)	0.5
Year after jail release	5 439 644	8398	154.39	6.85	23.3 (20.8-25.6)	4.4
Year in prison custody	1 322 335	323	24.45	1.09	0.9 (0.9-0.9)	1.1
Year after prison release	547 631	846	154.39	6.85	2.3 (2.1-2.6)	0.4
Age ≥18 y, no recent jail or prison	116 430 365	26 071	22.39	1.00	72.2 (61.3-79.0)	93.6
Age ≥18 y	124 363 710	36 113	29.04	1.29	100 (100-100)	100

Abbreviations: CMR, crude mortality rate; RR, relative risk.

^a^ Year after jail and prison release based on meta-analytic estimates from 6 studies.^16,18,20,21,22,23^ Data on people in jail were from Zeng and Minton^2^; data on people in prison (adjusted to include people in privately operated federal prisons) were from Substance Abuse and Mental Health Services Administration^8^ and Jones and Maynard^9^; and data on US population by age group were from Kinner et al.^10^

^b^ The RR was computed using the variable age 18 years or older, no recent jail or prison as the denominator.

^c^ Percentages of adult suicides and adults were computed from No. of adult suicides and No. of adults, respectively.

The RRs of suicide among US adults released from incarceration compared with those with no recent incarceration was 8.95 (95% CI, 7.21-10.69) in year 1 after jail release into the community (sensitivity analysis range, 8.43-11.09; ratio method estimate, 9.09 [95% CI, 5.48-13.47]) and 6.98 (95% CI, 4.21-9.76; ratio method estimate, 8.07 [95% CI, 5.25-10.90]) across 2 years after release (Table 3). Table 4 shows mean RRs of 25.13 (95% CI, 11.91-38.34) for females and 6.85 (95% CI, 3.05-10.65) for males. Although elevated, the 3.32 RR of suicide death in jail was lower than after release (Table 4).

People who were released from jail within the year accounted for an estimated 19.9% (95% CI, 16.2%-24.1%) of adult suicide deaths but only 2.8% of the adult population (Table 3) (sensitivity analysis range, 19.0%-23.4%; ratio method estimate, 20.2% [95% CI, 13.3%-27.0%]). The percentages for males and females were roughly equal (23.3% [95% CI, 20.8%-25.6%] and 24.0% [95% CI, 19.7%-36.8%]). The suicide RR was higher for females, but fewer females than males are incarcerated in jails.

Although suicide RR was highest in postrelease year 1, it was also high in postrelease year 2, when another 7.3% of all US adult suicides occurred. The SMR analysis indicated that 27.2% (95% CI, 18.0%-41.7%) of all US adult suicides occurred within 2 years after jail release (ratio method estimate, 30.1% [95% CI, 19.6%-40.7%]). Those in jail accounted for another 0.8% of adult suicides.

### Suicide Deaths in Prison and After Prison

People in or released from prison accounted for 2.3% (95% CI, 2.1%-2.6%) of suicide deaths. The suicide RR was 1.63 in state prison and 1.35 in federal prison (Table 4). The postrelease suicide SMR applied to persons released from both prison and jail. Since almost 12 times more people were released from jail (7 091 897) than from prison (603 844) annually, 1.7% (95% CI, 1.4%-2.0%) of adult suicide deaths occurred among people within the year of their prison release (sensitivity analysis range, 1.5%-2.0%); this group represented 0.2% of the US adult population in 2019 (Table 3). Adding the 19.9% estimate after jail release (Table 3), an estimated 21.6% of adult suicides (sensitivity analysis range, 20.6%-25.4%) occurred among the 3% of US adults within postrelease year 1 from jail or prison, with another 0.8% and 0.7% occurring during jail and prison incarceration, respectively (Table 4).

## Discussion

Suicide prevention efforts should focus on people who have spent at least 1 night in jail in the past year. Two distinct methods and datasets both indicate that during the year after jail release, an individual’s suicide CMR was 9 times the mean among other US adults, representing 19.9% of adult suicides in 2019, with 27.2% of suicides occurring within 2 years after release. Consistent with the high percentage of jail involvement among US adults who die by suicide, 35% of males and 13% of females who died by suicide in Denmark had gone to court for criminal charges^28^ and 25% of suicide decedents in Philadelphia had been in jail.^6^

The results suggest that better integration of suicide risk detection and prevention across health and criminal justice systems (including 911 calls, police contacts, pretrial jail detention, criminal courts, jail sentences, probation, and parole) is critical to advancing population-level suicide prevention efforts. However, high volumes of jail admissions and discharges, short jail stays, and understaffing mean that the 3119 local jails^2^ in the US generally have limited capacity to coordinate care with outside health agencies.^29^ Individuals are often arrested when experiencing acute mental health crisis (ie, manic or psychotic), arrests are typically unanticipated, jail stays are usually brief, and releases for individuals in pretrial jail detention are typically unscheduled. Community health systems are often unaware that their subscriber or patient was in jail and may drop them for missing appointments. The suicide rate after the return to the community after jail stay is higher than the suicide rate in jail, but local jails have limited capacity to coordinate postrelease health activities. Thus, a comprehensive approach to reducing the population-level US suicide rate would include health systems screening their subscribers or patients for recent arrest or police involvement and reaching out to those recently released to prevent suicide.^30,31,32^ In addition, broader use of 1115 Medicaid waivers to keep short incarcerations from disrupting Medicaid coverage could help to ensure that released individuals are insured, reducing barriers to health system reengagement.

It is now possible for health systems to link jail release data (which are typically publicly available) against their patient or plan participant list regularly and to systematize supportive outreach to patients who were recently released. Biomedical informatics advances have made searching text-based data from multiple jails in an area feasible and efficient. For example, CareSource, a large nonprofit managed care organization headquartered in Ohio, developed a data system that surveils booking and release from local jails across the US, identifies subscribers interacting with jails, and leverages that data for improved care coordination with community health care practitioners after release.^30^ This approach provides a potential solution to identifying a high-risk population for suicide that may not be well connected to standard care; the recently funded National Center for Health and Justice Integration for Suicide Prevention (NCHATS) is evaluating it.

Critical infrastructure problems have historically hindered evidence-based suicide prevention for recently incarcerated individuals. Justice settings often lack resources for needed suicide prevention interventions. Moreover, person-to-person rather than automated handoffs to health systems are currently not feasible at scale. The NCHATS is demonstrating that health systems can automate ethical, Privacy Rule–compliant identification of individuals being released from jails to share data with contracted community practitioners and provide outreach and care connection, solving this long-standing problem.^30^

Better epidemiologic studies are needed about people who pass through jail. The Bureau of Justice Statistics should identify the demographic distribution of people who are admitted or discharged and should test for demographic biases in length of jail stay. Deaths in a representative cohort also should be tracked for 2 years to validate the estimates in this study.

### Limitations

This analysis is subject to the limitations of the studies that we examined. Only 3 studies included death data beyond 2009. When meta-analyzing SMRs or CMR ratios, however, older data are problematic only if suicide patterns between groups diverge over time. After prison release, among the included 10 studies, suicide CMRs per 100 000 person-years ranged widely from 42.51 to 361.37. All studies ignored deaths that were coded as undetermined intent or unintentional drug overdoses, which were actually suicides or deaths of people who were ambivalent about dying; suicidality is common among drug overdose deaths.^33,34^ Available data on jail inmates were for the mean daily jail census. The daily census may not be representative of discharges; in large US jails, 5% of discharged individuals with stays longer than 180 days accounted for 41% of the daily census, whereas 62% of individuals discharged in less than a week accounted for 4%.^35^ Additionally, females made up 23.3% of jail admissions but only 14.7% of the daily census.^35^ In Ontario, unconvicted people or people released to community supervision on conviction accounted for 62.7% of persons released but 48.8% of the daily census.^17^ England and Wales^18^ as well as New Zealand^22^ experienced similar differentials. Burgeoning equity-driven reductions in cash bail are greatly decreasing the mean jail stay where implemented, potentially affecting suicide rates in jail and after release. We could not calculate CMRs by race and ethnicity because that information on people who were released from jail was unavailable and because reported US suicide CMRs by race and ethnicity are biased.^36^ Despite these limitations, the study findings were consistent across 2 methods and datasets and across 5 RR estimates with contrasting strengths and weaknesses.

## Conclusions

Adults released from jail make up a large, concentrated population at extreme risk for death by suicide. In this study, the nearly 7.1 million US adults released from jail in 2019 accounted for 1 in 5 adult suicides. Thus, identifying adults with recent jail releases can help with community suicide prevention efforts. Jails lack the staffing to manage an effective response to critical behavioral health needs after release. Health systems, including federally qualified health centers, are increasingly positioned to manage care coordination during high-risk transitions from jail to the community and should be recognized as important partners in building the cross-sector infrastructure necessary for identifying high-risk adults and providing comprehensive community-based suicide screening and prevention.

## Notes

Supplement 1. **eTable 1.** Multipliers and Calculations Used to Infer Suicide Counts in Years 1 and 2 After Release From Incarceration vs Counts for Other Periods **eTable 2.** Calculation of Crude Mortality Rate (CMR) for the Population Aged 18-68 in Minnesota Demographically Matched to Its Population of People Released From Prison in 2010-2019 **eTable 3.** Uncertainty Around Suicide Risk Estimates Over the Year and 2 Years After Release From Incarceration Compared to People Without Recent Incarceration **eTable 4.** Uncertainty Around Suicide Risk Estimates During Incarceration and Over the Year and 2 Years After Release From Incarceration Compared to People Who Are Incarcerated

Supplement 2.
Data Sharing Statement
