# Supplementary material for: Share of Adult Suicides After Recent Jail Release
Source: JAMA Netw Open. 2024 May 10;7(5):e249965. doi: 10.1001/jamanetworkopen.2024.9965 (PMC11087834; doi:10.1001/jamanetworkopen.2024.9965)
Supplement: Supplement 1. — eTable 1. Multipliers and Calculations Used to Infer Suicide Counts in Years 1 and 2 After Release From Incarceration vs Counts for Other Periods eTable 2. Calculation of Crude Mortality Rate (CMR) for the Population Aged 18-68 in Minnesota Demographically Matched to Its Population of People Released From Prison in 2010-2019 eTable 3. Uncertainty Around Suicide Risk Estimates Over the Year and 2 Years After Release From Incarceration Compared to People Without Recent Incarceration eTable 4. Uncertainty Around Suicide Risk Estimates During Incarceration and Over the Year and 2 Years After Release From Incarceration Compared to People Who Are Incarcerated [file jamanetwopen-e249965-s001.pdf]

Supplemental Online Content

Miller TR, Weinstock LM, Ahmedani BK, et al. Share of adult suicides after recent jail release. *JAMA Netw Open*. 2024;7(5):e249965. doi:10.1001/jamanetworkopen.2024.9965

eTable 1. Multipliers and Calculations Used to Infer Suicide Counts in Years 1 and 2 After Release From Incarceration vs Counts for Other Periods

eTable 2. Calculation of Crude Mortality Rate (CMR) for the Population Aged 18-68 in Minnesota Demographically Matched to Its Population of People Released From Prison in 2010-2019

eTable 3. Uncertainty Around Suicide Risk Estimates Over the Year and 2 Years After Release From Incarceration Compared to People Without Recent Incarceration

eTable 4. Uncertainty Around Suicide Risk Estimates During Incarceration and Over the Year and 2 Years After Release From Incarceration Compared to People Who Are Incarcerated

This supplemental material has been provided by the authors to give readers additional information about their work.

© 2024 Miller TR et al. *JAMA Network Open*

eTable 1. Multipliers and calculations used to infer suicide counts in years 1 and 2 after release from incarceration from counts for other time periods

Study	Ratio of suicides per 100,000 people in the 2 years vs 1 year after release from incarceration	Assumptions
Cunningham 2022[22]	1.34	None
Fitch 2023[23]	1.81	None
Haglund 2014[15]	1.42	None
Kariminia 2007[16]	1.34	CMR constant after mo 6
Kouyoumdjian 2016[17]	1.35	None
Pratt 2006[18]	1.60	CMR constant after wk 32
Spittal 2014[19] + Van Dooren 2013[21] [a]	1.57	CMR constant after y 1
Mean Ratio of 8 Studies	1.49	Unweighted average
Binswanger 2007[14] [b]	Unknown	CMR constant after y 1
McNeeley 2023[24] [c]	Unknown	CMR constant after y 1
Stewart 2004[20] [d]	Unknown	CMR constant after y 1

[a] Both studies report on the same cohort of releases. We calculated the year-2 rate by assuming a constant annual CMR after year 1. (371 suicides in 7.5 years – 84 suicides in year 1)/6.5 years = 44 suicides in year 2.

[b] Dividing the published suicide count at year 2 after release by the 1.49 mean ratio of counts through year 2 vs year 1 yielded the year-1 count in Table 1.

[c] Dividing the published 4.88-year count by 1.49 + 2.88 years X 0.49 yielded the year-1 count in Table 1. The cumulative year-2 count = 1.49 X the year-1 count.

[d] Dividing the published 3.4-year count by 1.49 + 1.4 years X 0.49 yielded the year-1 count in Table 1. The cumulative year-2 count = 1.49 X the year-1 count.

eTable 2. Calculation of Crude Mortality Rate (CMR) for the population aged 18-68 in Minnesota demographically matched to its population of people released from prison in 2010-2019

	Population	Suicides	CMR	Prisoners
Male	Ages 18-68			
Hispanic	87,8227	105	11.96	2,085.5
White Non-Hispanic	15,056,361	4,235	28.13	16,790.8
Black Non-Hispanic	1,096,966	180	16.41	9,495.9
Native Am	203,339	103	50.65	2,591.5
Asian	861,646	148	17.18	631.9
Race-weighted CMR	Male		25.17	31,595.5
Female	Ages 18-68			
Hispanic	774,083	19	2.45	277.5
White Non-Hispanic	14,957,074	1195	7.99	3,198.2
Black Non-Hispanic	1,041,188	37	3.55	606.1
Native Am	211,370	47	22.24	958.5
Asian	916,193	51	5.57	103.1
Race-weighted CMR	Female		9.77	5,143.5
Sex-Weighted CMR			23.01	36,739.0

Source: Population and suicide counts for Minnesota's general population ages 18-68 for 2010-2019 from WISQARS Fatal Injury online tabulation system. Prisoner counts by sex and race from Appendix A of McNeely (2018, reference 24).

© 2024 Miller TR et al. *JAMA Network Open*

eTable 3. Uncertainty around suicide risk estimates over the year and 2 years after release from incarceration compared to people without recent incarceration

SMR Released vs Gen'l Population	SMR over 1 year	SE	95% CI lower	95% CI upper	SMR over 2 years	SE	95% CI lower	95% CI upper	CMR over 1 year	SE	95% CI lower	95% CI upper	CMR over 2 years	SE	95% CI lower	95% CI upper
Binswanger, 2007	4.57	0.882	2.84	6.30	3.40	0.538	2.35	4.45	94.05	18.150	58.48	129.63	70.00	11.07	48.30	91.70
Cunningham, 2022	16.04	0.904	14.27	17.81	10.42	0.507	9.43	11.42	361.37	20.324	321.53	401.20	242.93	11.80	219.81	266.05
Fitch, 2023	1.94	0.144	1.66	2.22	1.93	0.106	1.72	2.13	42.51	3.159	36.32	48.70	42.75	2.36	38.12	47.38
Hagland, 2014	21.02	2.380	16.36	25.69	18.20	1.615	15.03	21.37	235.65	26.650	183.41	287.88	204.00	19.34	166.09	241.91
Kariminia, 2007	6.48	0.428	5.64	7.32	5.25	0.291	4.68	5.82	203.63	13.450	177.27	229.99	164.88	9.15	146.95	182.81
Kouyoumdjian, 2016	8.43	1.243	6.00	10.87	5.70	0.724	4.28	7.12	99.40	14.649	70.69	128.11	67.23	8.54	50.50	83.96
McNeeley, 2023	4.88	0.750	3.41	6.35	3.66	0.762	2.16	5.15	112.26	17.536	77.89	146.63	84.13	10.76	63.03	105.22
Pratt, 2006, corrected	8.92	0.456	8.02	9.81	7.13	0.288	6.56	7.69	155.93	7.971	140.30	171.55	124.66	5.04	114.78	134.54
Stewart, 2004	7.17	1.380	4.46	9.87	5.80	0.867	4.10	7.50	291.78	56.097	181.83	401.73	236.11	35.23	167.07	305.15
Van Dooren, 2013	12.00	1.309	9.43	14.57	9.60	0.848	7.93	11.26	216.67	23.615	170.38	262.95	173.01	15.28	143.06	202.95
10 studies combined	8.95	1.740	7.21	10.69	6.98	1.415	4.21	9.76	177.64	31.161	116.56	238.72	138.52	22.96	93.52	183.52

eTable 4. Uncertainty around suicide risk estimates during incarceration and over the year and 2 years after release from incarceration compared to people who are incarcerated

RR Released vs Inmates	RR 1 year	SE	95% CI lower	95% CI upper	RR over 2 years	SE	95% CI lower	95% CI upper	CMR inmates	SE	95% CI lower	95% CI upper
Binswanger, 2007	7.341	15.478	-23.00	37.68	5.464	8.209	-10.63	21.55	12.81	6.406	0.26	25.37
Cunningham, 2022	4.316	0.314	3.70	4.93	2.901	0.135	2.64	3.17	83.73	9.797	64.53	102.93
Fitch, 2023	4.337	0.842	2.69	5.99	4.362	0.805	2.79	5.94	9.80	1.941	6.00	13.60
Kariminia, 2007	1.626	0.030	1.57	1.69	1.317	0.018	1.28	1.35	125.22	10.551	104.54	145.90
Kouyoumdjian, 2016	2.043	0.276	1.50	2.58	1.382	0.116	1.16	1.61	48.65	10.251	28.56	68.74
McNeeley, 2023	8.797	8.336	-7.54	25.14	6.592	3.703	-0.67	13.85	12.76	3.684	5.54	19.98
Phillips 2019	2.554	0.025	2.51	2.60	1.981	0.019	1.94	2.02	83.00	1.757	79.56	86.44
Pratt, 2006, corrected	1.884	0.027	1.83	1.94	1.506	0.015	1.48	1.53	82.78	5.810	71.39	94.17
8 studies combined	2.700	0.467	1.78	3.31	2.122	0.379	1.38	2.87	56.84	15.145	27.16	86.52

© 2024 Miller TR et al. *JAMA Network Open*
