# Supplementary material for: Share of Adult Suicides After Recent Jail Release
Source: JAMA Netw Open. 2024 May 10;7(5):e249965. doi: 10.1001/jamanetworkopen.2024.9965 (PMC11087834; doi:10.1001/jamanetworkopen.2024.9965)
Supplement: Supplement 2. — Data Sharing Statement [file jamanetwopen-e249965-s002.pdf]

Data Sharing Statement

Miller. Share of Adult Suicides After Recent Jail Release. *JAMA Netw Open*. Published May 10, 2024. doi:10.1001/jamanetworkopen.2024.9965

Data
Data available: No

Additional Information
Explanation for why data not available: We did not collect patient data. This is a modelling exercise using data from two meta-analyses we conducted as input. All already-aggregated data we used are tabulated in the manuscript.
